# Diversity in approach to teaching and assessing ethics education for medical undergraduates: A scoping review

**DOI:** 10.1016/j.amsu.2020.06.028

**Published:** 2020-06-27

**Authors:** Anne D Souza, Vina Vaswani

**Affiliations:** a Kasturba Medical College, Manipal, Manipal Academy of Higher Education, Manipal, 576104, India; b Head of Forensic Medicine Department, Centre for Ethics, Yenepoya Medical College, Yenepoya (Deemed to Be University), Deralakatte, Mangaluru, India

**Keywords:** Ethics education, Medical undergraduates, Teaching methods, Assessment, Curriculum, Review

## Abstract

There are diverse methods to teach medical ethics, and there is no single accepted approach towards its learning and assessment. The authors aim to explore the various strategies practised to teach undergraduate medical students the fundamentals of medical ethics and their evaluation. The authors reviewed the articles published from January 2014 to September 2019. The authors searched PubMed for the relevant publications and extracted the information using a data extraction sheet. Twenty-nine articles were included for the review, which fulfilled the inclusion criteria. Case-based discussions were a widely accepted strategy to learn ethics. The studies highlighted a mixed teaching approach using multiple teaching tools. A qualitative approach was preferred for the assessment through reflections, simulated patient interactions, and development of portfolios. However, there are gaps in the existing literature on the assessment strategies for ethics education. Heterogeneity still exists in the planning of the curricula, teaching, and assessment methods. These curricula suit the cultural and religious set up of that particular country. Case-based discussion is a popular teaching strategy, and there exist numerous innovative and cost-effective active teaching strategies. There is a need for studies that are more rigorous to address the evaluation of the ethics curricula. This review would help educators to choose their preferred approach based on their teaching environment.

## Highlights

• The major learning strategies were ‘Case-based,’ ‘video/movies,’ ‘role modelling,’ ‘lectures,’ and ‘patient interactions.
• A mixture of learning methods have proven to be beneficial.
• The assessment of ethics education is still a challenge, and there is a gap in the literature on their strategies.
• Curricula are evaluated mainly by the student feedback using unstructured, open-ended questionnaires and reflections.
• There is a need to have studies that are more rigorous to evaluate the long-term impact of the ethics curricula.

## 1 Introduction

Medical ethics is a system of moral principles that apply values and judgments to the practise of medicine [1]. Knowledge of medical ethics would aid a physician in making decisions during the care they provide with due consideration to ethical principles [2].

The Hippocratic oath has highlighted the relationship between medicine and ethics during ancient times [3]. However, the present-day situation has called for efforts to incorporate ethics into the medical curriculum d/discipline-based, community-based/hospital-based, [2,[4], [5], [6]]. SPICES model (student-centered/teacher-centered, problem-based/information gathering, integrate elective/uniform and systematic/apprenticeship based) of curriculum plan proposed by Harden et al. is one of the oldest models and is one of the foundations for learning and assessing ethics teaching modules in the medical curriculum [7].

In 2012, the Medical Council of India (MCI) proposed guidelines for professional conduct, etiquette, and ethics for the practising doctors [8]. It was followed by the introduction of AETCOM (Attitude, Ethics, and Communication) module in 2015 that played a significant role in implementing ethics in the undergraduate medical curriculum. Under the umbrella of AETCOM, elements such as fundamentals of bioethics, communication skills, medico-legal issues, and patient-doctor relationship were to be included in the medical curriculum [9].

There is a wide range of strategies used to teach ethics in medical education. Problem-based learning (PBL) and case-based discussions are highly effective, but their long term effectiveness is debated [[10], [11], [12]].

Several reviews in the literature explore ethics education in the past [[13], [14], [15]]. Eckles et al. in their report, have highlighted the deficits in the literature on the teaching methods and measuring effectiveness in ethics education [14]. Therefore, this review, while aiming to explore the different existing strategies practised in recent years by medical schools to teach their students the fundamentals of medical ethics and their assessment, intended to identify the current gaps in the literature. The study aims to identify the recent trends using the research question ‘What are the diverse methods to teach and assess medical ethics for undergraduate medical students, and how their outcome is evaluated?’

## 2 Methodology

The articles published during the last five years from January 2014 to September 2019, were reviewed in October 2019. We searched PubMed by building a search strategy using MeSH key terms' ethics', ‘medical ethics,’ ‘medical students,’ ‘education,’ ‘teaching,’ ‘techniques,’ ‘activities’ (Annexure 1). AD performed the initial search using the search strategy. At first, all the search results were screened for their titles and abstracts and selected the articles for full-text screening. Both the authors (AD & VV) then separately read the full texts of the selected articles and included the relevant publications for the final review. We scrutinized the reference lists of each included articles and added the related articles.

### 2.1 Inclusion and exclusion criteria

We included the studies published in the English language on teaching and assessing ethics to undergraduate medical students (first year to internship), which included research reports, viewpoints, and letters to the editor. We excluded the studies conducted only on research ethics, articles dealing with ethics in postgraduate medical education, and practising doctors and the unpublished data, such as conference presentations. We constructed a review protocol (Annexure 1) and presented the report according to the existing guidelines to conduct scoping reviews [16].

### 2.2 Extraction of data

The authors extracted the data using the data extraction sheet in excel format (Annexure 1). Demographic details (authors, place and year of work, country) and the details of the study design, teaching and assessment methods, highlights, outcome, etc. were extracted.

### 2.3 Summarizing and reporting

We tabulated the demographic details of the studies and described the different teaching and assessment methods used in each study. We identified the studies carried out to develop a curriculum for ethics education and discussed the ways of measurement of their outcomes.

We reviewed the articles qualitatively and coded for elements such as teaching methods, assessment methods, and outcome/evaluation. We identified the themes related to the different approaches in teaching and assessing medical ethics.

## 3 Results

**Fig. 1:**
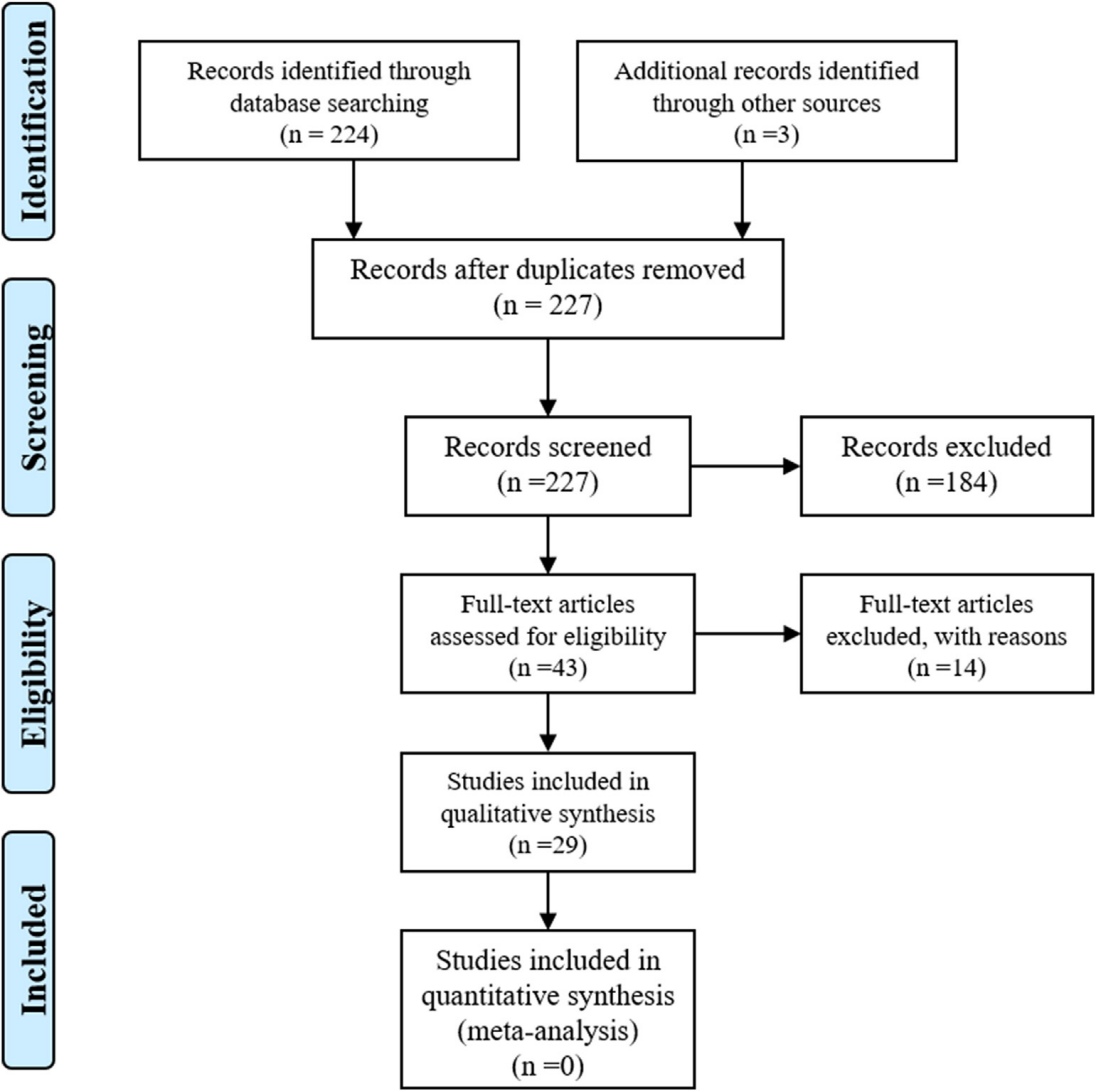
PRISMA flow diagram for the article selection process. The author has taken this flow diagram from the already available literature (Aromataris and Riitano) [43].

The first search in PubMed resulted in 224 articles matching the search criteria. Title and abstract screening of these resulted in the selection of 40 publications for the full-text review. The authors reviewed all 40 full-text articles and considered 26 articles for the final report. From the reference list of these 26 articles, three more articles met the criteria to be included. We considered 29 publications for the final reporting. The Preferred Reporting Items for Systematic Reviews and Meta-Analyses (PRISMA) flow diagram depicts the details of the search process (Fig. 1).

### 3.1 Demographic description of the studies

Of the 29 studies reviewed, there were 11 studies from the USA, nine from Europe, four from Asian countries, three from the UK, one from Africa and one from Canada. Of the 29 studies selected, 12 studies were published between 2014–15, and the remaining 17 were published between 2016–19. The studies were cross-sectional (eight), perspectives (seven), and research reports (five). Annexure 2 shows the detailed demographic picture of the studies.

### 3.2 Teaching methodologies for ethics

Themes were recognised from the studies to identify the teaching methods. Though they addressed similar topics of medical ethics, the teaching methods were heterogeneous. ‘Case-based discussions,’ ‘video/movies,’ ‘role modelling,’ ‘interactive lectures,’ and ‘patient interactions’ were construed as emerging themes.

The usage of case-based discussion as a teaching strategy was found in 15 studies. However, the formulation and the usage of cases was remarkably heterogeneous. A case-based approach was one of the powerful learning tools, considering the adequate preparations, and the active discussion with the facilitator [17]. The concept of peer teaching was an easy and effective method for teaching ethics [18]. In a study, there was a collection of medical genetics ethics cases constructed incorporating the essential topics [19]. In one study, there was an interprofessional approach practised, and the trained faculty facilitated the case-based discussion session [20]. Table 1 provides additional information on the case based discussion approach.

**Table 1:** Description of the different approaches towards case-based discussions.

S. No.	Authors	Number of students trained	The topic for the cases	Set-up
1	Cheung [29]	Not specified	End of life communication & care	Not specified
2	Aguilera et al. [30]	73 over four years	Moral status, patient autonomy, informed consent; nonresuscitation orders, futility, advanced directives; ethical decision-making; confidentiality privacy; research versus practice; professionalism; ethical priority setting in health care	Longitudinal over six years of undergraduate period
3	Schildmann et al. [26]	28 institutes of Germany (100–350 students per year)	End of life care, patient autonomy, the beginning of life and research ethics (Top four topics)	Incorporated in the regular curriculum (N = 19) or as a separate model curriculum (N = 5) or a parallel curriculum (N = 4)
4	Mahajan et al. [31]	17 interns (pilot run)	Group dynamics; medical oaths; MCI and other regulations; principles of ethics, rights, and duties of patients and doctor; legal and ethical issues in body and organ donation, abortion, MTP, informed consent; Confidentiality, medical negligence, Euthanasia; Conflict of interest, Ethical dilemmas, and their resolution; Communication skills	Four-day long workshop conducted for the interns
5	Vergano et al. [4]	Not specified	Clinical ethics and end-of-life care	One day-long interactive session
6	Goldberg et al. [20]	Final year students of 2015 (N = 28) and 2016 (N = 56)	Palliative care ethics	A four-week module on palliative care ethics. One hour weekly session
7	Dasgupta [19]	180 first-year medical students	Direct-to-consumer genetic testing, patient privacy, economic and legal issues of genetic testing	Two-hour session
8	Robeson & King [28]	Not specified	Bioethics & clinical ethics	Performable case study model

Streaming of videos and movies that emphasise an ethical value were found to be used as learning tools in six studies. Schochow & Steger in Germany surveyed the utility of the e-learning platform for the construction of multimedia tools and resources in medical ethics, history, and medical terminology in 31 different educational institutes [21]. Vergano et al. introduced a course of medical ethics for the critical care curriculum. The course had interactive training, clinical cases, videos, role-playing, small group discussions, and exercises [4]. Movies can address general, deontological, and distinctive ethical issues, principles of bioethics, and theories of ethics [22]. In a prospective cohort study, a cohort of medical students watched movies on topics such as truthtelling, gender ethics, contraception, and lack of autonomy. The students reflected the importance of the course through a semi-structured interview [23].

‘Role modelling’ emerged as a theme in four studies, and ‘interaction with the patients’ in two studies. A questionnaire-based study described ‘role modelling’ as one of the excellent learning tools. The students strongly opined that the interactions with the patients and incorporation of ethical issues during teaching rounds help in a better understanding of ethics [6]. A report by Papanikitas et al. revealed the importance of interaction and peer support in ethics education [24]. A survey conducted in Poland and the USA by Makowska reported that growing up with a physician in the family would create an impact on the medical students concerning their interactions with the pharmaceutical agencies [25].

Four studies highlighted the importance of ‘interactive lectures’ in ethics education. Schildmann et al. in their survey across the German medical faculties, revealed lectures as one of the commonly used learning tools [26]. The combined practise of didactic lectures, case discussions, and a component of simulation would make ethics education work [27]. The use of theatre in medical ethics was one of the rarest but thought-provoking learning tools that were encountered. At Wake Forest University, the students in small groups first discuss and analyze the contextual material and characters, then formulate research to build the case and script the case in groups, and the post-performance discussion with the facilitator promoted learning [28].

### 3.3 Assessment of ethics education

Of the 29 studies reviewed, only 11 studies had emphasised on the assessment strategies for ethics education. Table 2 provides a comprehensive view of the different assessment tools practised.

**Table 2:** The studies depicting the different assessment tools in ethics education.

Serial No.	Authors	Country	Assessment strategy
1	Giugliani et al. [45]	Brazil	Student feedback. Consulting the families of the patients for their satisfaction with the resident's ethical conduct
2	Schildmann et al. [26]	Germany	Multiple-choice exams, assignments, presentations, Single exam with open questions
3	Schochow & Steger [21]	Germany	e-exams
4	Mahajan et al. [2]	India	Theory questions in the university examination should test knowledge competencies. Skill competencies assessed by clinical, practical, and viva.
5	Goldberg et al. [20]	United Kingdom	Student reflections at the end of the course
6	Ferreira-Padilla et al. [5]	Spain	Students' work during their internship Practical test, OSCE, the students' attendance, active participation, and the portfolio
7	Ekmekçi [38]	Turkey	Written exam
8	Bilgin et al. [39]	Turkey	Assignments to be assessed
9	AlMahmoud et al. [6]	UAE	Staff observation during clinical supervision, simulated patient interactions, oral examination for knowledge assessment. For the skill evaluation, direct observation of the students by the faculty during their actual interaction with the patients. Patient evaluation of the students.
10	Miranda & Sanchez [44]	USA	Reviewing the essays written by the students, student participation, and discussion. Case scenarios to assess the application of ethics
11	Bosch-Barrera et al. [12]	Spain	A continuous and final assessment of PBL cases by the faculty

### 3.4 Curriculum development and evaluation

Of the 29 studies, nine studies had curricula formulated for teaching ethics. Cheung developed a curriculum using the Structured Learning in Clinical Ethics (SLICE) model for the respiratory residency program. The module addressed the end of life care and its ethical values. The students read assignments and actively participated in case-based discussions. The residents felt more at ease in handling the end of life situations, and the faculty who taught this curriculum said this module had reformed their attitude [29].

Trained faculty conducted case-based active learning workshops for undergraduate students in a Practical Curriculum in Clinical Ethics (PRACTICE) curriculum proposed by Aguilera et al. It had introduced a new pedagogical approach. It provided opportunities for new ethics faculty to gain experience in both subject material and content delivery [30].

An initiative to conduct faculty orientation workshops was taken by Smith in 2014, to facilitate and train the faculty involved in teaching ethics. The seminar titled ‘Ethics across the curriculum’ or ‘ethics boot camps,’ organised for the teachers involved in teaching ethics gave the faculty orientation, understanding and hands-on experience of how to conduct ethics classes [17].

Module for interns in Medical Ethics (MIME) developed by Mahajan et al. for the medical interns proposed a curricular pattern. The interns took this 18-h course through mixed learning strategies like games, interactive lectures, case-based discussions, role play, and cinema [31].

Students’ Medical Ethics Rounds (SMER) was a 3-h session proposed by Beigy et al. During this, the expert faculty addressed topics like confidentiality and honesty, medical team errors, informed consent, medical education ethics, conflicts of interest and end of life issues [32].

Goldberg et al. developed a four-week module on palliative care ethics (PCE) titled Acting Internship in Critical Care (AICC) for final year medical students implemented by an interprofessional faculty team. A student and faculty guide was provided as a resource material, containing the outline and structure of 1-h rounds and questions for facilitating the session. Students reported a better understanding of end of life care at the end of the rotation [20].

Simulation as a core strategy has transformed ethics education. Tritrakarn et al. introduced simulation-based clinical scenarios using various teaching tools such as manikins, task trainers, standardised patients, or role-play by staff, or students are often practised [27].

Biomedical Ethics and Humanities Scholarly Concentration (BEHM SC) was a unique curriculum developed by Liu et al. in which the students shadowed the ethics consults and attended ethics committee meetings. The students had to undertake a scholarly project required for their graduation [33]. Ethical Life Support (ELS) by Vergano et al. was a curriculum developed to sensitise the students towards the ethical issues in critical care. Airway–Breathing–Circulation–Disability sequence was converted into an Acknowledge–Be aware–Communicate–Deal approach [4].

These curricula are the result of tremendous effort and needed curriculum evaluation for their further improvement. Table 3 provides a summary of the curriculum evaluation of these modules.

**Table 3:** Summary of ethics curricula and strategies for their evaluation.

S. No.	Authors	Country	Module	Curriculum evaluation
1	Cheung [29]	Canada	SLICE (Structured Learning in Clinical Ethics)	Student feedback
2	Aguilera et al. [30]	Central America	PRACTICE curriculum	Student feedback
3	Smith [17]	USA	EAC (Ethics Across the Curriculum)	Attendee satisfaction through a questionnaire
4	Mahajan et al. [31]	India	MIME (Module for interns in medical ethics)	Feedback questionnaire
5	Beigy et al. [32]	Iran	SMER (named Students' Medical Ethics Rounds)	Pretest-posttest score analysis
6	Goldberg et al. [20]	United Kingdom	AICC (Acting Internship in Critical Care)	Student feedback
7	Tritrakarn et al. [27]	USA	Simulation-based teaching	Advised student feedback
8	Liu et al. [33]	USA	BEHM SC (Biomedical Ethics and Humanities Scholarly Concentration)	Interviewing the graduates retrospectively
9	Vergano et al. [4]	Italy	ELS (Ethical Life Support)	Student feedback

## 4 Discussion

Teaching ethics in undergraduate medical education is an integral part of the medical curriculum across the world. However, it is still sporadic when it comes to developing a curriculum. The bulk of the literature was from the USA and Europe, indicating that they have implemented ethics in the undergraduate medical curriculum with clear objectives and outcomes, as seen in 23 out of the 29 studies reviewed. The surveys from the USA addressed the teaching and assessment strategies elaborately and explained how the medical schools had implemented them as curricula for undergraduate medical students [17,20,27,29,30]. Ethics education curricula reviewed by Dubois in 2002 revealed that the ethics education was far from homogeneous among U.S. medical schools, in both content and extensiveness. The authors tried to demonstrate the significant areas of overlap to come up with one ideal ethics curriculum [34].

Several authors have reviewed the ethics education in the past [13,14,35]. Goldie, in 2000, discussed the ethics curricula and proposed a systematic plan to develop an ethics curriculum for medical undergraduates [13]. Apart from the case-based teaching, problem-based and team-based approaches were the commonly used teaching methods in the past [10,36]. Currently, the ethics curriculum exists in many medical schools. However, there exists a gap in the assessment methodologies and evaluating the long-term effectiveness of ethics education. Not all institutes who adapted ethics education assessed it.

The ethics curricula of medical schools address the concept of medical ethics keeping in mind the cultural values of that particular country [31]. These curricula included a wide range of topics, from ethical principles to end of life care [4,26,31].

There were nationwide surveys that revealed the current trend of ethics education in medical schools. Of the 44 medical schools in Spain, the authors compared the ethics curriculum between private and public schools, recently founded and the older schools. The number of credits for ethics was two times higher in newer schools when compared to the older ones. Only 1/5th of schools evaluated the ethics curriculum through practical application [5]. Schildmann et al. identified the courses related to the history, theory, and ethics of medicine in Germany [26]. Such surveys could provide a broader picture of the current trend in ethics education.

Case-based discussions were the widely accepted strategy to learn ethics [4,5,[17], [18], [19], [20],^24^,^26^,^27^,[29], [30], [31],[37], [38], [39]]. There have been different approaches to cased based discussions. The case discussions allow students to participate actively and help them understand better. The discussions would enhance students’ capability to handle such situations in their later practise.

Bebeau, in her research report, opines that problem based practice (using cases) can be especially useful in helping students recognise and subsequently avoid personal interest arguments while conducting research [40]. Structured feedback should follow the case discussion, which would help students to build ethical reasoning [41].

Several studies highlighted a mixed teaching approach using multiple teaching tools for ethics [2,[4], [5], [6],^12^,^21^,^23^,^24^,^26^,[30], [31], [32], [33],^39^]. However, lectures were one of the least used strategies. Lecturing, when kept short and interactive, has a benefit of making the students understand the concepts [26,27,31,39].

There is still a scarcity in the existing literature on the assessment strategies for ethics education. The studies, which assessed the students, used more of a qualitative approach such as reflections, simulated patient interactions, and development of portfolios [5,6,20,29]. Beigy et al. in their study, said that assessing students for their change of attitude was one of their challenges that needs further exploration [32]. There is a need to develop effective assessment strategies for ethics that would not only evaluate the knowledge and skills attained but the impact of their ethics education in their actual practise.

The articles reviewed revealed two types of curricula. The first type was an ethics curriculum for the medical undergraduates of first to final year [17,[29], [30], [31],^33^]. The second type addressed only particular aspects of medical ethics like the end of life care, critical care, and clinical ethics [4,20]. A comprehensive approach to the curriculum would give a broader picture to the students, and the subject-specific curricula would make them correctly understand the ethical issues that would help them handle such situations in real-life practise.

Timely evaluation of the formed curricula is essential to understand its impact. A study by Goldie et al. has elaborated on the importance of curriculum evaluation for an ethics curriculum [42]. The current review revealed immediate student feedback as the dominant strategy adopted for curriculum evaluation [4,17,[29], [30], [31]]. Liu et al. in their study, interviewed the graduates retrospectively on their experience of learning ethics and their current views [33]. Such an approach seems to have a better outcome for long-term evaluation of ethics curricula.

## 5 Limitations

This review highlighted the teaching and assessment strategies in undergraduate medical education. The authors found the studies conducted worldwide, but there were still countries from which such literature is still lacking. The included studies were heterogeneous in their design, and the majority were of cross-sectional in design. The risk of bias would be one of the confounding factors while assessing the long-term impact of ethics education. We also agree that our search was not so rigorous, as we had excluded the grey literature.

## 6 Conclusion

A defined curriculum in ethics exists in medical schools that follow a longitudinal pattern in teaching ethics to the medical undergraduates. Heterogeneity still exists in the planning of the curricula, teaching, and assessment methods. These curricula suit the cultural and religious set up of that particular country. Although case-based discussion is a well-known teaching strategy, there exist numerous innovative and cost-effective active teaching strategies. Knowledge of these strategies would help educators to choose their preferred approach based on their teaching environment. The assessment of ethics education is still a challenge, and there is a gap in the literature on their strategies. The studies, which assessed the students, used more of a qualitative approach such as reflections, simulated patient interactions, and development of portfolios. Most of the studies evaluated the ethics curricula mainly by the student feedback using unstructured, open-ended questionnaires, and reflective writing. Only one study used a retrospective approach by interviewing the graduates on their learning experience and practise. Such an approach would be better in evaluating the long-term impact of ethics education. To address this, we need to have studies that are more rigorous to assess the long-term effect of the ethics curricula.

## Ethical approval

The Institutional Ethics Committee has exempted the study from the ethics review.

## Sources of funding

This study did not receive any funding from external agencies.

## Author contribution

Anne D Souza & Vina Vaswani together conceptualized the study. Anne D Souza performed the initial search through PubMed. Both the authors separately then scrutinized the articles for inclusion in the review. Anne D Souza prepared the draft manuscript and Vina Vaswani critically reviewed it.

Anne D Souza as a requirement for the completion of Postgraduate Diploma in Bioethics & Medical Ethics during December 2019 with the guidance of Vina Vaswani presented the initial work.

## Trial registry number

As the current work is a scoping review, as per guidelines it has not been registered.

1. Name of the registry:

2. Unique Identifying number or registration ID:

3. Hyperlink to your specific registration (must be publicly accessible and will be checked):

## Guarantor

1. Dr. Anne D Souza.

2. Dr. Vina Vaswani.

## Declaration of competing interest

The authors state that there is no conflict of interest to declare.

## Figures and Tables

**Table:** 

Title of the article			
Authors			
Journal			
Year of publication			
Place of research			
Type of the study			
Teaching methods used			
Assessment methods used			
Major outcomes			
Highlights of the study			
Limitations			

**Table:** 

S. No.	Authors	Year of publication	Country	Study design
1	Smith [17]	2014	Clemson, USA	Perspective
2	Bebeau [40]	2014	Minneapolis, USA	Perspective
3	Herried [37]	2014	New York, USA	Perspective
4	Marshall [18]	2014	Phoenix, USA	Perspective
5	Papanikitas et al. [24]	2014	United Kingdom	Conference report
6	Tritrakarn et al. [27]	2014	USA	Perspective
7	Miranda & Sanchez [44]	2014	USA	Research report
8	Aleksandrova-Yankulovska [21]	2014	Bulgaria	Survey
9	Giugliani et al. [14]	2015	Brazil	Research report
10	Schochow & Steger [21]	2015	Germany	Questionnaire-based cross-sectional study
11	Wintrup [46]	2015	UK	Debate
12	Bosch-Barrera et al. [12]	2015	Spain	Research report
13	Mahajan et al. [2]	2016	India	Perspective (curriculum development)
14	Beigy et al. [32]	2016	Iran	Cross-sectional (curriculum development)
15	Ferreira-Padilla et al. [5]	2016	Spain	Observational, descriptive-comparative, and transverse study
16	Ekmekçi [38]	2016	Turkey	Narrative review
17	Greenberg et al. [22]	2016	Canada	Prospective Cohort
18	Schildmann et al. [26]	2017	Germany	Survey
19	Cheung [29]	2017	Canada, USA	Perspective
20	Mahajan et al. [31]	2017	India	Pilot study on curriculum innovation
21	Makowska [25]	2017	Poland	Questionnaire-based cross-sectional study
22	AlMahmoud et al. [6]	2017	UAE	Questionnaire-based cross-sectional study
23	Dasgupta [19]	2017	USA	Research report (curriculum development)
24	Robeson & King [28]	2017	USA	Concept paper
25	Goldberg et al. [20]	2018	Northwell, UK	Cross-sectional (Curriculum planning & evaluation)
26	Bilgin et al. [39]	2018	Turkey	Cross-sectional (Qualitative study)
27	Liu et al. [33]	2018	USA	Retrospective, qualitative study
28	Aguilera et al. [30]	2019	Central America	Case study
29	Vergano et al. [4]	2019	Italy	Editorial (curriculum development)

## ANNEXURE 1 PROTOCOL

### Background

Medical education has evolved over the past decade, and there has been an emphasis on competency-based approach. This approach demands students to have a knowledge of ethics, humanities, and attitudinal values at the early phase of their medical school. Now, there are diverse methods for teaching medical ethics, and there is no single accepted approach towards its learning and assessment. Therefore, this review aims to explore the various strategies practiced by medical schools to teach their students the fundamentals of medical ethics and their evaluation.

### Objectives

The current review aims to.

• Identify the different approaches of teaching ethics for medical undergraduates
• Identify the various strategies of assessment for ethics for medical undergraduates
• Analyze the effectiveness of different attitudes towards training and assessing ethics for medical undergraduates

### Methods

This review would include the articles published from January 2014 to September 2019. PUBMED will be the database to search for the relevant items. The grey literature, including conference proceedings and unpublished material, will also be accessed. The search strategy includes the Mesh key terms. We will categorize our search in three stages that involve searching for the keywords in titles, in abstracts, and from the reference list of the obtained literature.

#### Inclusion and exclusion criteria

We will include the studies addressing the ethics education to the medical undergraduates from first to final year, interns. We will consider the articles published in the English language only. We will also include the research reports, viewpoints, letters to the editor. However, we wish to exclude the studies conducted only on research ethics. The search will not be limited to certain countries but across the globe. We will eliminate the articles dealing with ethics in postgraduate medical education and practicing doctors.

The author would follow the guidelines for conducting a scoping review [16] to carry out this research work.

#### Search strategy

((((ethics [Title] OR medical ethics [Title]) AND ((“students, medical" [MeSH Terms] OR (“students" [All Fields] AND “medical" [All Fields]) OR “medical students" [All Fields] OR (“medical" [All Fields] AND “students" [All Fields])) OR (medical [All Fields] AND undergraduates [All Fields]))) AND ((((“education" [Subheading] OR “education" [All Fields] OR “teaching" [All Fields] OR “teaching" [MeSH Terms]) OR (“teaching" [MeSH Terms] OR “teaching" [All Fields] OR (“educational" [All Fields] AND “techniques" [All Fields]) OR “educational techniques" [All Fields])) OR (“teaching" [MeSH Terms] OR “teaching" [All Fields] OR (“teaching" [All Fields] AND “methods" [All Fields]) OR “teaching methods" [All Fields])) OR (“teaching" [MeSH Terms] OR “teaching" [All Fields] OR (“training" [All Fields] AND “activities" [All Fields]) OR “training activities" [All Fields]))) AND (“2014/01/01" [PubDate]: “2019/09/31" [PubDate]))

#### Extraction of data

The authors will extract the data using the data extraction sheet constructed based on the guidelines given by Peters et al. [16]. We will include the demographic details (authors, place and year of work, country) and the details on the study design, teaching and assessment methods, highlights, outcome, etc. We will save the data in excel format for constructing the results.

#### Summarizing and reporting the results

The authors will describe the demographic details of the studies based on the form of a table. Further, we will describe the different teaching and assessment methods used in each study. At the later stage, we will highlight the studies carried out to develop a learning module for ethics in medical education. We will then describe how they had measured their outcomes.

## Footnotes

Appendix A Supplementary data to this article can be found online at https://doi.org/10.1016/j.amsu.2020.06.028.

## References

[1] Munyaradzi M. (2012). Critical reflections on the principle of beneficence in biomedicine. Pan Afr Med J.

[2] Mahajan R., Aruldhas B.W., Sharma M., Badyal D.K., Singh T. (2016). Professionalism and ethics: a proposed curriculum for undergraduates. Int J Appl Basic Med Res.

[3] Emery A.E. (2013 Nov 1). Hippocrates and the oath. J. Med. Biogr..

[4] Vergano M., Naretto G., Elia F. (2019). ELS (Ethical Life Support): a new teaching tool for medical ethics. Crit Care.

[5] Ferreira-Padilla G., Ferrández-Antón T., Lolas-Stepke F., Almeida-Cabrera R., Brunet J., Bosch-Barrera J. (2016 Oct). Ethics competences in the undergraduate medical education curriculum: the Spanish experience. Croat. Med. J..

[6] AlMahmoud T., Hashim M.J., Elzubeir M.A., Branicki F. (2017). Ethics teaching in a medical education environment: preferences for diversity of learning and assessment methods. Med Educ.

[7] Harden R.M., Sowden S., Dunn W.R. (1984). Educational strategies in curriculum development: the SPICES model. Med. Educ..

[8] (2002 Sep). The Indian Medical Council (professional conduct, etiquette and ethics) regulations, 2002. Issues Med Ethics.

[9] Mitra J., Saha I. (2016 Apr 1). Attitude and communication module in medical curriculum: rationality and challenges. Indian J. Publ. Health.

[10] Heidari A., Adeli S.H., Taziki S.A. (2013). Teaching medical ethics: problem-based learning or small group discussion?. Journal of Medical Ethics and History of Medicine.

[11] Tysinger J.W., Klonis L.K., Sadler J.Z., Wagner J.M. (1997 Oct). Teaching ethics using small-group, problem-based learning. J. Med. Ethics.

[12] Bosch-Barrera J., Briceño García H.C., Capella D., De Castro Vila C., Farrés R., Quintanas A. (2015 Aug). [Teaching bioethics to students of medicine with problem-based learning (PBL)]. Cuad Bioet Rev Of Asoc Espanola Bioet Etica Medica.

[13] Goldie J. (2000 Feb). Review of ethics curricula in undergraduate medical education. Med. Educ..

[14] Eckles R.E., Meslin E.M., Gaffney M., Helft P.R. (2005 Dec). Medical ethics education: where are we? Where should we be going? A review. Acad Med J Assoc Am Med Coll.

[15] de la Garza S., Phuoc V., Throneberry S., Blumenthal-Barby J., McCullough L., Coverdale J. (2017 Aug). Teaching medical ethics in graduate and undergraduate medical education: a systematic review of effectiveness. Acad. Psychiatr..

[16] Peters M.D.J., Godfrey C.M., Khalil H., McInerney P., Parker D., Soares C.B. (2015 Sep). Guidance for conducting systematic scoping reviews. Int. J. Evid. Base. Healthc..

[17] Smith K.C. (2014 Dec 15). Ethics is not rocket science: how to have ethical discussions in your science class. J. Microbiol. Biol. Educ..

[18] Marshall P.A. (2014 Dec 15). Integrating ethics into case study assignments. J. Microbiol. Biol. Educ..

[19] Dasgupta S. (2017). Medical Genetics Ethics Case Collection: Discussion Materials for Medical Students in the Genomic Era. MedEdPORTAL.

[20] Goldberg G.R., Weiner J., Fornari A., Pearlman R.E., Farina G.A. (2018). Incorporation of an Interprofessional Palliative Care-Ethics Experience Into a Required Critical Care Acting Internship. MedEdPORTAL.

[21] Schochow M., Steger F. (2015). State of Digital Education Options in the areas of Medical Terminology and the History, Theory and Ethics of Medicine. GMS Z Med Ausbild.

[22] Aleksandrova-Yankulovska S. (2016 Mar). An innovative approach to teaching bioethics in management of healthcare. Nurs. Ethics.

[23] Greenberg R.A., Kim C., Stolte H., Hellmann J., Shaul R.Z., Valani R. (2016 Jul 27). Developing a bioethics curriculum for medical students from divergent geo-political regions. BMC Med. Educ..

[24] Papanikitas A., Spicer J., McKenzie-Edwards E., Misselbrook D. (2014). 4th annual primary care ethics conference: ethics education and lifelong learning. Lond. J. Prim. Care.

[25] Makowska M. (2017). Does growing up with a physician influence the ethics of medical students' relationships with the pharmaceutical industry? The cases of the US and Poland. BMC Med Ethics.

[26] Schildmann J., Bruns F., Hess V., Vollmann J. (2017). "History, Theory and Ethics of Medicine": The Last Ten Years. A Survey of Course Content, Methods and Structural Preconditions at Twenty-nine German Medical Faculties. GMS J Med Educ.

[27] Tritrakarn P., Berg B.W., Kasuya R.T., Sakai D.H. (2014 Aug). Medical school hotline. Hawai‘i J. Med. Public Health.

[28] Robeson R., King N.M.P. (2017). Performable Case Studies in Ethics Education. Healthcare (Basel).

[29] Cheung L. (2017 Apr 25). Creating an ethics curriculum using a structured framework. Int. J. Med. Educ..

[30] Aguilera M.L., Martínez Siekavizza S., Barchi F. (2019 Jan-Dec). A Practical Approach to Clinical Ethics Education for Undergraduate Medical Students: A Case Study From Guatemala. Journal of Medical Education and Curricular Development.

[31] Mahajan R., Goyal P.K., Sidhu T.K., Kaur U., Kaur S., Gupta V. (2017 Dec). Module for interns in medical ethics: a developmental diegesis. Int J Appl Basic Med Res.

[32] Beigy M., Pishgahi G., Moghaddas F. (2016). Students' medical ethics rounds: a combinatorial program for medical ethics education. J Med Ethics Hist Med.

[33] Liu E.Y., Batten J.N., Merrell S.B., Shafer A. (2018). The long-term impact of a comprehensive scholarly concentration program in biomedical ethics and medical humanities. BMC Med Educ.

[34] DuBois J.M., Burkemper J. (2002 May). Ethics education in U.S. medical schools: a study of syllabi. Acad Med J Assoc Am Med Coll.

[35] Mulhearn T.J., Steele L.M., Watts L.L., Medeiros K.E., Mumford M.D., Connelly S. (2017). Review of instructional approaches in ethics education. Sci. Eng. Ethics.

[36] Chung E.-K., Rhee J.-A., Baik Y.-H., A O-S (2009 Jan 1). The effect of team-based learning in medical ethics education. Med. Teach..

[37] Herreid C.F. (2014 Dec 15). Cautionary tales: ethics and case studies in science. J. Microbiol. Biol. Educ..

[38] Ekmekçi P.E. (2016). Medical ethics education in Turkey; state of play and challenges. Int Online J Educ Teach.

[39] Bilgin A.C., Timbil S., Guvercin C.H., Ozan S., Semin S. (2018 Jun). Preclinical students' views on medical ethics education: a focus group study in Turkey. Acta Bioeth.

[40] Bebeau M.J. (2014 Dec 15). An evidence-based guide for ethics instruction. J. Microbiol. Biol. Educ..

[41] Smith S., Fryer-Edwards K., Diekema D.S., Braddock C.H. (2004 Mar). Finding effective strategies for teaching ethics: a comparison trial of two interventions. Acad Med J Assoc Am Med Coll.

[42] Goldie J., Schwartz L., Morrison J. (2000). A process evaluation of medical ethics education in the first year of a new medical curriculum. Med. Educ..

[43] Aromataris E., Riitano D. (2014 May). Constructing a search strategy and searching for evidence. A guide to the literature search for a systematic review. Am. J. Nurs..

[44] Miranda D., Sanchez D.J. (2014 Dec 15). The tuskegee experiment: an introduction in ethics for pre-healthcare professional students. J. Microbiol. Biol. Educ..

[45] Giugliani R., Baldo G., Vairo F., Lujan Lopez M., Matte U. (2015 Jul). The Latin American School of Human and Medical Genetics: promoting education and collaboration in genetics and ethics applied to health sciences across the continent. J Community Genet.

[46] Wintrup J. (2015). The changing landscape of care: does ethics education have a new role to play in health practice?. BMC Med Ethics.

